# Specific and Nonuniform Brain States during Cold Perception in Mice

**DOI:** 10.1523/JNEUROSCI.0909-23.2023

**Published:** 2024-01-05

**Authors:** Haritha Koorliyil, Jacobo Sitt, Isabelle Rivals, Yushan Liu, Adrien Bertolo, Silvia Cazzanelli, Alexandre Dizeux, Thomas Deffieux, Mickael Tanter, Sophie Pezet

**Affiliations:** ^1^Physics for Medicine Paris, INSERM, ESPCI Paris, CNRS, PSL Research University, Paris 70015, France; ^2^PICNIC Lab, Inserm U 1127, ICM, Institut du Cerveau et de la Moelle épinière, Paris F-75013, France; ^3^Equipe de Statistique Appliquée, ESPCI Paris, PSL Research University, UMRS 1158, Paris 75005, France; ^4^Iconeus, Paris 75014, France

**Keywords:** Doppler, functional connectivity, primary sensory cortex, thermal sensitivity, ultrafast ultrasound imaging

## Abstract

The quest to decode the complex supraspinal mechanisms that integrate cutaneous thermal information in the central system is still ongoing. The dorsal horn of the spinal cord is the first hub that encodes thermal input which is then transmitted to brain regions via the spinothalamic and thalamocortical pathways. So far, our knowledge about the strength of the interplay between the brain regions during thermal processing is limited. To address this question, we imaged the brains of adult awake male mice in resting state using functional ultrasound imaging during plantar exposure to constant and varying temperatures. Our study reveals for the first time the following: (1) a dichotomy in the response of the somatomotor–cingulate cortices and the hypothalamus, which was never described before, due to the lack of appropriate tools to study such regions with both good spatial and temporal resolutions. (2) We infer that cingulate areas may be involved in the affective responses to temperature changes. (3) Colder temperatures (ramped down) reinforce the disconnection between the somatomotor–cingulate and hypothalamus networks. (4) Finally, we also confirm the existence in the mouse brain of a brain mode characterized by low cognitive strength present more frequently at resting neutral temperature. The present study points toward the existence of a common hub between somatomotor and cingulate regions, whereas hypothalamus functions are related to a secondary network.

## Significance Statement

Very little is known regarding the brain areas involved in thermal coding and the interactions between them. Using an emerging neuroimaging technique in freely moving mice during exposure to either constant neutral/warm or cold sensation or varying temperature, this study identified dynamic brain states. Some are observed more frequently during exposure to cold stimuli. Through the demonstration of reproducible and specific fingerprints of dynamic brain modes, this study will open the path to future investigations on the alterations of these brain states during cold sensing in animal models and human subjects affected by peripheral neuropathy.

## Introduction

Thermal sensation and perception are crucial for maintaining the structural and functional integrity of all organisms (Gracheva and Bagriantsev, 2015). Thermal changes can elicit a multitude of responses including rapid motor withdrawal reflex and thermoregulation to maintain core body temperature. To cope with the changes in the thermal environment, physiological and behavioral mechanisms are employed permanently (Tan and Knight, 2018). The complex mechanisms that result in the central and peripheral integration of cutaneous thermal sensations is still not completely understood.

Thermal sensations felt on skin are encoded and transmitted to the central nervous system by primary sensory neurons, such as non-myelinated C fibers and thinly myelinated Aδ fibers whose terminals act as free nerve endings on the skin (Middleton et al., 2021; Xiao and Xu, 2021). The thermal information is transmitted by the sensory afferents to the dorsal horn via the TRP channels, which are the molecular thermodetectors (Peier et al., 2002; Clapham, 2003; Patapoutian et al., 2003; Bandell et al., 2004; Bautista et al., 2007; Dhaka et al., 2007; Tan and McNaughton, 2016; Hoffstaetter et al., 2018; Vandewauw et al., 2018; Vilar et al., 2020) and can initiate well-defined responsive pathways, such as the following: (1) activation of motor neurons resulting in a rapid withdrawal reflex, (2) transmission of thermal information via the spinothalamic tract to various nuclei of the thalamus and finally to several cortical areas such as the insular cortex and the primary somatosensory cortex where it takes the form of a perceived temperature, (3) initiation of thermoregulatory responses (Vriens et al., 2014). Although the supraspinal pathways work synergistically to form a thermal perception, the complex interplay among them is less explored. Studies have shown that several thalamic nuclei (Bushnell et al., 1993; Duncan et al., 1993; Craig et al., 1994; Davis et al., 1999), somatosensory regions (Becerra et al., 1999; Moulton et al., 2012; Milenkovic et al., 2014), and the insula (Craig et al., 2000; Olausson et al., 2005; Veldhuijzen et al., 2010; Peltz et al., 2011; Wager et al., 2013; Gogolla et al., 2014) are crucial for thermosensation. Although the cingulate region is not a direct part of the thermosensory circuit, it is involved in the affective responses to the nociceptive thermal stimulations (Vogt, 2005). The preoptic anterior hypothalamus (POAH) has been linked to thermoregulatory behavior in numerous studies (Ishiwata et al., 2002; DiMicco and Zaretsky, 2007; Wang et al., 2019).

The present study aimed at understanding the involvement of some of the aforementioned brain regions using functional ultrasound (fUS) imaging, which is a relatively new versatile neuroimaging modality that allows imaging and measurement of cerebral blood volume (CBV) in humans (Demene et al., 2017; Imbault et al., 2017; Soloukey et al., 2020), nonhuman primates (Dizeux et al., 2019), and rodents (Macé et al., 2011; Sieu et al., 2015; Urban et al., 2015; Bergel et al., 2018; Rahal et al., 2020) with excellent spatial (100–300 µm) and temporal resolutions (down to 20 ms). One of its most important characteristics is its high sensitivity compared with fMRI (Boido et al., 2019). Indeed, during a task, due to neurovascular coupling, the locally increased neuronal activity leads to a strong hemodynamic response (Iadecola, 2017). In the past, fUS imaging proved sensitive enough to enable the measurement of the cortical hemodynamic changes induced by sensory (Macé et al., 2011), olfactory (Osmanski et al., 2014a), and visual (Macé et al., 2018) stimuli in anesthetized animals. Another very important characteristic of fUS imaging consists in its ability to perform acquisitions in awake and behaving animals (Montaldo et al., 2022), as demonstrated for auditory stimuli in awake animals (Bimbard et al., 2018) or motor tasks (Sieu et al., 2015; Bergel et al., 2020). Taking advantage of the sensitivity of this technique (Osmanski et al., 2014b) and the ability to study freely moving animal, this study aimed at improving our understanding of the processing of warm and cold sensing by studying the changes of intrinsic brain connectivity in freely moving mice during plantar exposure to warm, cold, and neutral surfaces and to the variations of the surface temperature. Due to the heavy weight of ultrasound probes required to perform 3D imaging, and to the role of the primary sensory cortex in cold processing, this study used a 2D linear probe centered on the part of the primary sensory cortex corresponding to the hindlimb and on the hypothalamus. The analysis of the static and dynamic functional connectivity (FC) reveals that cold induces a strongly increased connectivity in the somatomotor (SM) network but also a decreased connectivity between the SM areas and the hypothalamus.

## Materials and Methods

### Animals

The experiments were conducted in compliance with the European Community Council Directive of 22 September 2010 (010/63/UE) and the local ethics committee (Comité d'éthique en matière d'expérimentation animale No. 59, “Paris Centre et Sud,” project 2018-05). Accordingly, the number of animals in our study was kept to the minimum necessary. Due to previous studies using a similar experimental design (Rabut et al., 2020), we established that *N* = 6 animals per group was the smallest number of animals required to detect statistically significant differences in our imaging experiments. Using the intra- and interanimal variability established by Rabut et al. (2020) and the variations of static functional connectivity measured in the various experimental groups in two preliminary experiments (testing the effect of fixed temperatures and of ramps of temperatures, respectively), we performed a G-Power calculation (https://www.psychologie.hhu.de/arbeitsgruppen/allgemeine-psychologie-und-arbeitspsychologie/gpower) and concluded that between *N* = 8 and *N* = 6 animals per group, respectively, were necessary. Finally, all methods are in accordance with ARRIVE guidelines.

Animals arrived at the animal facilities 1 week before the beginning of experiments. Fourteen C57Bl/6 male mice (aged 7 weeks at the beginning of the experiments) were obtained from Janvier Labs (France) and housed under controlled temperature (22 ± 1°C), relative humidity (55 ± 10%), with a 12 h light/dark cycle. Finally, food and water were available ad libitum. Experiments typically lasted for 4–6 weeks.

**Figure 1.**
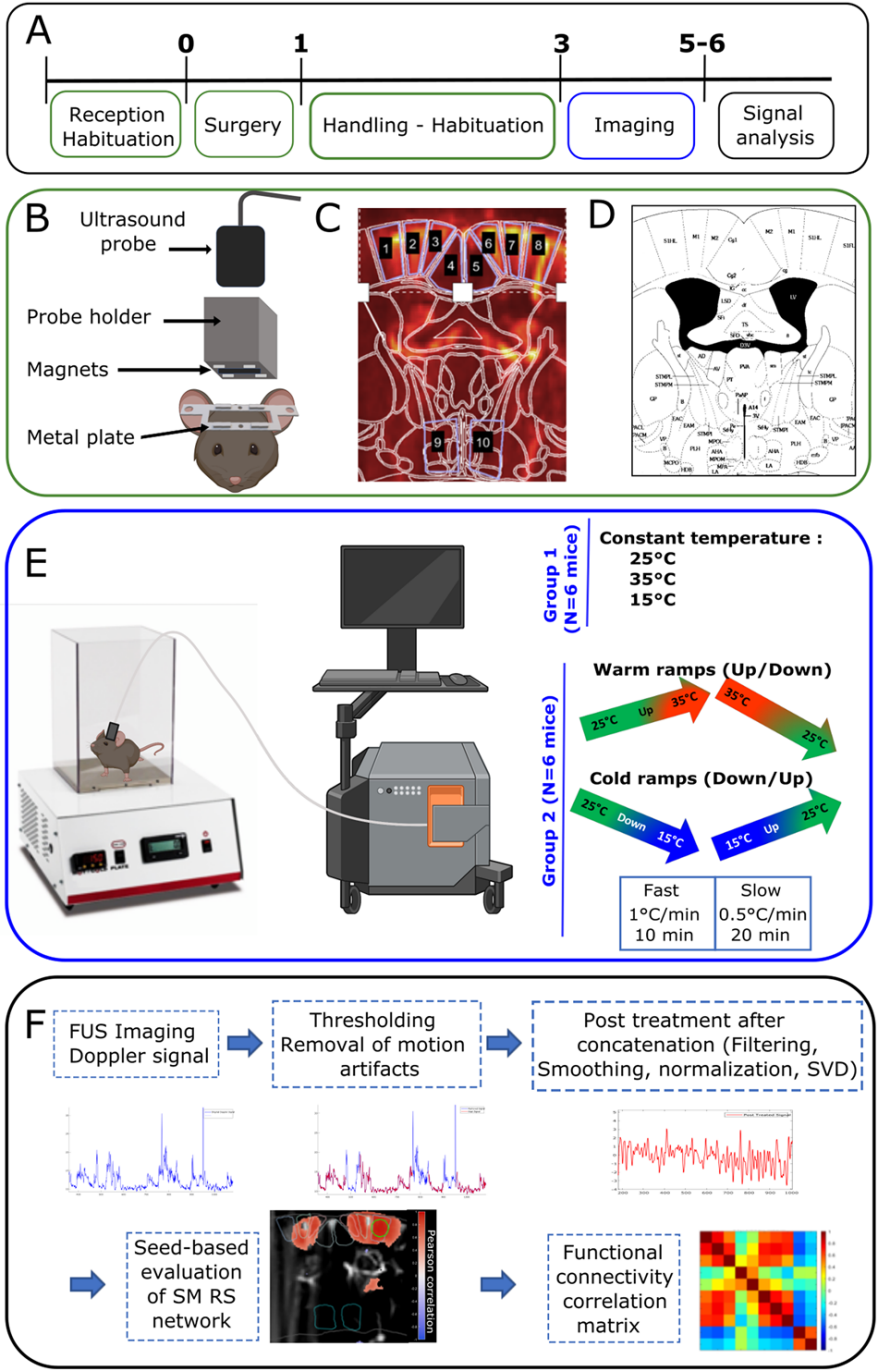
Experimental design and timeline of the experiments. ***A***, One week after their arrival in the laboratory, a metal plate was surgically attached to the mice skulls. After 1 week of recovery, mice were handled and habituated for 2 weeks. Imaging was then performed for the next 2 weeks or more, depending on the quality of the skull and on the well-being of the mice. ***B***, Schematic showing the metal plate, probe holder, fUS probe, and (***C***, ***D***) plane of imaging (bregma −0.34 mm). ***C***, Doppler image superimposed with the delimitation of the mouse brain atlas (***D***; Paxinos and Franklin, 2012). The regions of interest are as follows: 1,8 primary somatosensory cortex, hindlimb part; 2,3,6,7 primary and secondary motor cortices; 4,5 cingulate cortex; and 9,10 Hypothalamus. ***E***, Experimental setup with Bioseb and fUS imaging (Iconeus). Mice were exposed to constant temperatures or to warm and cold ramps at fast or slow pace. ***F***, The Doppler signal obtained from imaging underwent thresholding to remove motion artifacts, concatenation, low-pass filtering, smoothing, normalization, and SVD filtering. The cleaned Doppler signal was then used for FC analysis, for which a first test using a seed-based analysis of the strength of the SM resting state FC network was verified, followed then by static and dynamic analyses of the FC. The images in panels ***E*** and ***F*** were modified from Biorender (https://app.biorender.com/). Extended Data Figure 1-1 is reporting the identity of the mice included in each set of experiments, as well as the number of imaging sessions kept per animal in our analysis.

Constant temperature and temperature ramp experiments were conducted in two different sets of *N* = 8 and *N* = 6 mice (Fig. 1). When possible, animals were imaged more than once in each experimental condition. Indeed, due to motion artifacts, some sessions had to be discarded (see below). Details of data included from the various animals are listed in Extended Data Figure 1-1.

10.1523/JNEUROSCI.0909-23.2023.f1-1 Figure 1-1 Tables reporting the identity of the mice included in each set of experiments, as well as the number of imaging sessions kept per animal in our analysis. Download Figure 1-1, PDF file.

### Experimental design

The global aim of this study was to decipher how thermal sensations are encoded in the mouse brain. As intrinsic functional connectivity was shown to measure the activity and functionality of the brain networks, we postulated that it could vary during exposure to cold, warm, and neutral floor surfaces. To address this issue, we imaged the brain using fUS in awake and freely moving mice exposed to various thermal sensations (Fig. 1*E*).

We observed in previous experiments (Rabut et al., 2020) that motion artifacts deeply alter the quality of the ultrasound signals and that all efforts need to be made to prevent these artifacts. In preliminary experiments, we have sought to establish the range of temperature in which the animals were minimally uncomfortable. Combining the records of the animal's natural behavior (grooming, exploration, urination, and freezing) and measurements of naturally emitted ultrasound vocalizations, we observed that, between 15 and 35°C, the animals were minimally uncomfortable and the experiments feasible. Any residual of motion artifacts in the Doppler signal (due to head movements or behavioral movements such as grooming or licking) were removed using a dedicated signal processing, as illustrated in Figure 1*F*. As is described below, the data kept come from periods when the animals were quiet, in resting state.

We used the Bioseb “Hot-Cold Plate” to conduct the experiments. The metal floor of the Bioseb equipment can be kept at a constant temperature or heated or cooled down at different rates.

#### Constant temperature

A constant floor temperature of 15°C (cold), 25°C (neutral), or 35°C (warm) was applied for 20 min. Experiments at these temperatures were randomly repeated on 2 separate days. At the beginning of all sessions, the floor temperature was held at 25°C, and the transition to the desired temperature occurred within seconds.

#### Varying temperature at fast or slow pace

Floor temperature variations in the warm domain were made of two ramps, one up (from 25 to 35°C) and one down (back to 25°C); variations in the cold domain were made of a ramp down (from 25 to 15°C) and a ramp up (back to 25°C; Fig. 1*E*). In order to determine the effect of the speed of the temperature change, these ramps were performed at two different rates: either at 0.5°C per minute (for 20 min) or at 1°C per minute (for 10 min). The mice were reimaged at least three times. In order to avoid any bias due to the order of these variations, the order of warm and cold variations was randomized. They are denoted as follows:

Cool ramps

1. CFD, cool fast down: 25 to 15°C (−1°C per minute)
2. CFU, cool fast up: 15 to 25° (+1°C per minute)
3. CSD, cool slow down: 25 to 15°C (+0.5°C per minute)
4. CSU, cool slow up: 15 to 25°C (−0.5°C per minute)

Warm ramps

1. WFU, warm fast up: 25 to 35°C (+1°C per minute)
2. WFD, warm fast down: 35 to 25°C (−1°C per minute)
3. WSU, warm slow up: 25 to 35°C (+0.5°C per minute)
4. WSD, warm slow down: 35 to 25°C (−0.5°C per minute)

### Surgical implantation of metal plate

Approximately 1 week after their arrival, the mice underwent surgery for the implantation of the metal plate (Tiran et al., 2017; Rabut et al., 2020). A mixture of ketamine (100 mg/kg) and medetomidine (1 mg/kg) was administered intraperitoneally, and then the mouse was placed on a stereotaxic frame where the skull bone was exposed after skin and periosteum removal. The metal plate was fixed on the skull using Super-Bond C&B (Sun Medical), and small screws were minimally drilled in the skull. The field of interest was ∼5 mm wide, between the bregma and lambda points. The surgery took 45–60 min to be completed. Subcutaneous injections of atipamezole (1 mg/kg, antisedan) and metacam (5 mg/kg/d) were given to reverse the anesthesia and to prevent postsurgical pain, respectively. A protective cap was mounted on the metal plate using magnets to protect the skull and to keep the field of imaging intact for 4–6 weeks (Bertolo et al., 2021). Altogether, the metal plate and the cap did not interfere with the normal daily activity of the mice. After a recovery period of 1 week, the mice proceeded toward the habituation phase.

### Habituation and training

After recovering from the metal plate implantation, the mice were subjected to an extensive habituation protocol. To make sure that the mice were not under any stress during the experiment, it was crucial that they were at ease with the user and the setup. They were initially handled by the user and then exposed to the Bioseb HC Plate. Their daily interaction time in the Bioseb apparatus was gradually increased from 15 to 30 min, 1 h, 1 h 30 min and finally 2 h. Depending on the level of habituation of each mouse, the user practiced protective cap removal, skull cleaning using saline, and application of echographic gel without anesthesia, by gently restricting the head movement. After each session, the mice received a reward. The process lasted 2 weeks, and, depending upon the comfort level of the mice, we then proceeded to the imaging phase.

### Transcranial awake fUS imaging

Three days prior to the first imaging session, the mice were anesthetized with isoflurane (1.5%). The respective probe holders were magnetically clipped to the metal plate. Real-time transcranial Doppler images were acquired using the NeuroScan acquisition software (Inserm Technology Research Accelerators and Iconeus), and the position of the probe was adjusted to select the bregma −0.34 mm plane. The regions of interest (ROIs) included the primary somatosensory cortex of the hindlimb, the primary and the secondary motor cortex, the cingulate cortex, and the hypothalamus (Fig. 1*C*,*D*). The skull was then thoroughly inspected and cleaned to avoid any infection. The mice were put back in their cages and imaged only 3 d later to avoid any interference with the isoflurane anesthesia.

Unlike the previous awake imaging protocols described in Tiran et al. (2017), mice were not anesthetized to prepare the skull during the imaging phase. They were trained and accustomed to the detachment of the protective cap, to the cleaning of the skull with saline, and to the application of echographic gel with minimal force. They were then gently introduced into the Bioseb apparatus and the probe holder was attached to the implanted metal frame using the magnets on both pieces. Experiments began shortly after.

Real-time vascular images were obtained by ultrafast compound Doppler imaging technique (Deffieux et al., 2021). Eleven successive tilted plane waves (−10° to +10° with 2° steps) were used for insonification. Each image was obtained from 200 compounded frames acquired at 500 Hz frame rate corresponding to a 5.5 kHz pulse repetition frequency. The tissue signal was isolated from the cerebral blood volume signal using a spatiotemporal clutter filter based on the singular value decomposition (SVD) of raw ultrasonic data (Demene et al., 2015) to obtain a power Doppler image.

### Doppler signal analysis

Imaging in awake mice required careful removal of motion artifacts due to head movements or behavioral movements such as grooming. We followed the analysis previously described in Rabut et al. (2020) by first using a SVD clutter filter to separate blood motion from tissue motion and then by thresholding tissue motion and Doppler signal to identify the frames with motion artifacts. Several thresholds were investigated by carefully examining the tissue motion signal, and the threshold that removed most of the motion artifacts was chosen. We kept and concatenated epochs of at least 50 consecutive time points. The concatenated cleaned frames were filtered using a low-pass filter with a cutoff frequency of 0.1 Hz to extract the steady state. A polynomial fit of order 3 was applied to detrend the signal, and, finally, global variations in the brain were suppressed by removing the first eigenvector of the dataset before connectivity analysis (Fig. 1*F*). Acquisitions that did not match the aforementioned criteria (<50 consecutive time points) were discarded. Extended Data Figure 1-1 summarizes the identity of animals included in both parts of the study and how many sessions from each mouse were kept. Out of the *N* = 8 animals included in the constant experiments, *N* = 8 acquisitions per experimental conditions were used in the analysis. As for the second part of the study (ramp experiments), they included 6–10 acquisitions (Extended Data Fig. 1-1), obtained in *N* = 6 mice. Comparison of the movies performed during the experiments (using a CCD camera) and the time points kept by our algorithm showed that all time points where the animals were walking and grooming were excluded. The data kept were from the period when the animals were quiet, in resting state.

### Static/dynamic functional connectivity characterization and respective statistical analyses

In agreement with previous studies that described strong interhemispheric connectivity using a seed-based approach (Pais-Roldán et al., 2021), we delineated manually a seed in the somatosensory network and observed a strong correlation of signals both in the ipsilateral SM resting state network, but also in the contralateral SM cortex (Fig. 1*F*). Due to the caveat in studying FC using the seed-based approach in experimental groups of animals (Preti et al., 2017), we used the definition of regions of interest, with the mouse Paxinos Atlas (Paxinos and Franklin, 2012) and used two complementary approaches to study functional connectivity: static and dynamic FC analyses.

Unlike the conventional analysis of time-averaged FC that provides quantitative information on the correlation (or anticorrelation) between pairs of ROIs in the steady state, there have been tremendous advancements in the study of the dynamic nature of FC (Hutchison et al., 2013; Preti et al., 2017). The temporal evolution of FC can reveal how FC reshapes according to the physiological (Tarun et al., 2021) or behavioral changes, at rest, or during a task, or in case of neurodegenerative or neuropsychiatric illnesses (Tian et al., 2018; Demertzi et al., 2019; Gu et al., 2020).

In the two types of analyses, the following brain regions of the imaging plane were studied: Primary somatosensory hind limb part, primary and secondary motor, cingulate, and hypothalamus (Fig. 1*C*,*D*). This imaging plane was chosen because of the known role of some of these regions in thermosensation and thermoregulation. Ten ROIs were defined based on the Paxinos Atlas, numbered and coded as follows:

1. 1 (S1HLL) and 8 (S1HLR): primary somatosensory cortex left and right
2. 2 (M1L) and 7 (M1R): primary motor cortex left and right
3. 3 (M2L) and 6 (M2R): secondary motor cortex left and right
4. 4 (CgL) and 5 (CgR): cingulate cortex left and right
5. 9 (HyThL) and 10 (HyThR): hypothalamus left and right

#### Static FC analysis

In order to study the resting-state FC in the different thermal conditions, the post-treated time course of the CBV signal of the *N* = 10 ROIs (Fig. 1*C*,*D*) were extracted. Simply stated, the temporal signal during the calm periods (without motion artifacts) was extracted from each ROI. The *N* × *N* Pearson correlation matrix of the ROI signals was computed over time. First, each correlation coefficient of the correlation matrix was individually compared between pairs of conditions. Since the measurements were not always independent (because of intra- and intergroup comparison, and of repeated measurements on some animals, see Extended Data Fig. 1-1), these comparisons were made with linear mixed models having the animal as random effect factor and the thermal condition as two-modality fixed effect factor, whose significance was tested. The correlation coefficients being Fisher transformed, the parameter estimation was made using restricted maximum likelihood estimation, and the validity of the model was checked posteriori by testing the normality of its residuals with Shapiro–Wilk's test. Then, in order to account for multiple testing [a *N* × *N* correlation matrix involves *N* × (*N*−1) / 2 = 45 correlation coefficients], we performed Benjamini–Hochberg's adjustment for multiple comparisons on the *p* values of significance of the thermal condition effect. A false discovery rate of 0.05 was adopted.

#### Dynamic FC analysis

In order to study the dynamic behavior of FC, the above correlation matrices can be decomposed into the contribution of each time point, that is, the cofluctuation matrices (Esfahlani et al., 2020). As a matter of fact, consider ***x****_i_* = [*x_i_*(1) … *x_i_*(*T*)]*^T^* the time series of the CBV signal of ROI *n*°*i*, and ***x****_j_* = [*x_j_*(1) … *x_j_*(*N*)]*^T^* that of ROI *n*°*j*. On the one hand, the correlation coefficient between ROIs *n*°*i* and *j* can be computed by first *z*-scoring each ***x***_i_ according to ***z****_i_* = (***x****_i_* − *m_i_*) / *s_i_*, where *m_i_* = 1 / *T* ∑*_t_ x_i_*(*t*) and *s_i_*^2^ = 1 / (*T*−1) ∑*_t_* (***x****_i_* − *m_i_*)^2^ are the empiric mean and variance of the time series over time, and then by computing *r_ij_* = ***z****_i_^T^* ***z****_j_* / (*T* − 1). Hence a single *N* × *N* correlation matrix with only *N* × (*N* − 1) / 2 = 45 elements of interest were computed since the matrix is symmetric with unit diagonal. On the other hand, it is possible to consider the element-wise product of ***z****_i_* and ***z****_j_* as encoding the magnitude of the moment-to-moment cofluctuations between ROIs *n*°*i* and *j*, and the 3D array of the *N* × *N* couples of ***z****_i_* and ***z****_j_* as a time series of *T* cofluctutation matrices of size *N* × *N*, each with only *N* × (*N* + 1) / 2 = 55 elements of interest since the cofluctuation matrices are symmetric.

To assess the repetitive nature of the dynamic characteristics of brain networks as a response to thermal inputs in an unsupervised fashion, we performed *k*-means clustering of the cofluctuation matrices contributing to the static correlation matrices. The time series data from all 92 acquisitions on all animals were concatenated together to form a single time series of *T* = 84,499 time points for each ROI, yielding a 3D array of size 84,499 × 10 × 10.

Prior to *k*-means clustering, outliers were removed. To this end, the 84,499 × 84,499 L1 distance matrix between cofluctuation matrices considered as *N* × (*N* + 1) / 2 vectors was computed, and the matrices with mean *z*-scored distance to the others larger than 3 were discarded, decreasing to *T*’ = 83,325 the number of cofluctuation matrices to be clustered. *K*-means clustering was performed on the T’ remaining matrices considered as *N* × (*N* + 1) / 2 vectors using the L1 distance with *K* = 7 clusters. We also chose this number of clusters for its optimality according to Duda and Hart's criterium (Duda et al., 1973). Briefly, Duda and Hart's criterium for *K* clusters is the number of clusters among the *K* that should be split into two, according to appropriate tests of the significance of the corresponding intraclass variance reductions. This number drops to zero for *K* = 7 and remains equal to zero for larger *K*.

**Figure 2.**
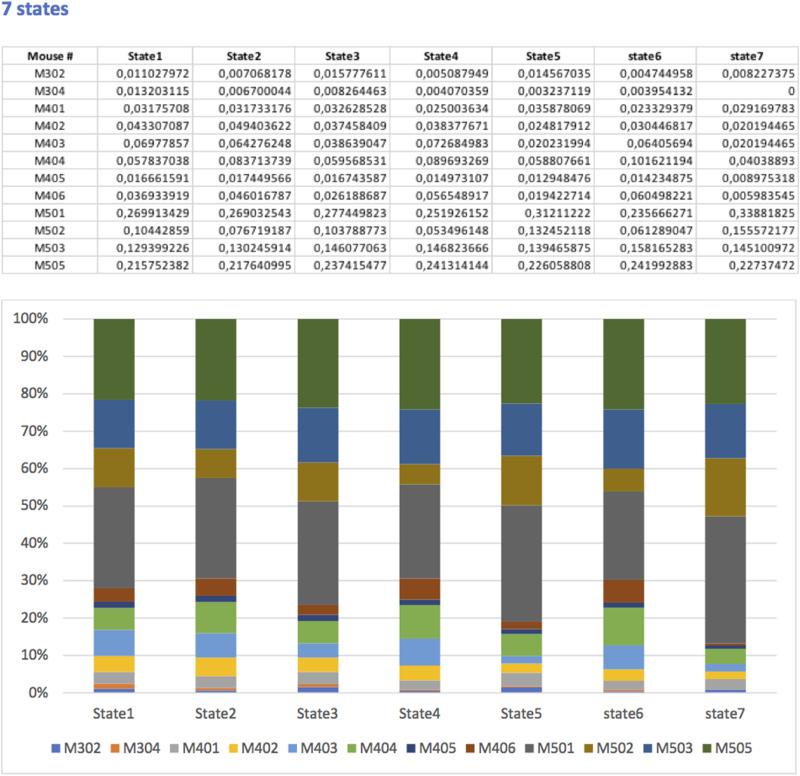
Distribution of the different animals in the different brain states.

To avoid local minima, the algorithm was run 500 times with random initializations of centroid positions, the configuration minimizing the total sum of intracluster distances being retained. Finally, each time point was assigned to a cluster (or brain state), resulting in a dynamic characterization of FC patterns. The occurrence rates of all brain states were calculated for each animal in all conditions. Prior to the comparison of these occurrence rates across thermal conditions, we checked the homogeneity of the animal distribution across the brain states (Fig. 2). In order to establish the significance of the effect of the thermal condition on the occurrence rate of each state, linear mixed models were used for the same reasons as for the elements of the correlation matrices (intra- and intergroup comparison, varying numbers of repeated measurements). As before, the animal was considered as a random effect factor, and the thermal condition as a fixed effect factor, this time with several modalities (the constant and varying temperature conditions). When the latter could be considered significant with a type I error risk of 5%, two-by-two comparisons were performed, and the corresponding *p* values were adjusted using Benjamini–Hochberg's procedure, a false discovery rate of 0.05 being again adopted.

### Data availability

Source data are available on the repository website Dryad using the following link: https://doi.org/10.5061/dryad.mkkwh713t.

### Code availability

The classical codes used to generate the results are available in the following depository: https://doi.org/10.5061/dryad.mkkwh713t. The custom codes used for the analysis of fUS data used in this study are protected by INSERM.

## Results

### Changes in brain functional connectivity during exposure to sustained neutral/warm or cold floor surface

In order to decipher the FC alterations in brain networks, which are indicators of dynamic changes in the brain, we developed an experimental setup based on a previously established protocol on awake and freely moving mice (Rabut et al., 2020), but this time allowing the imaging during the application of varying floor temperatures (Fig. 1).

**Figure 3.**
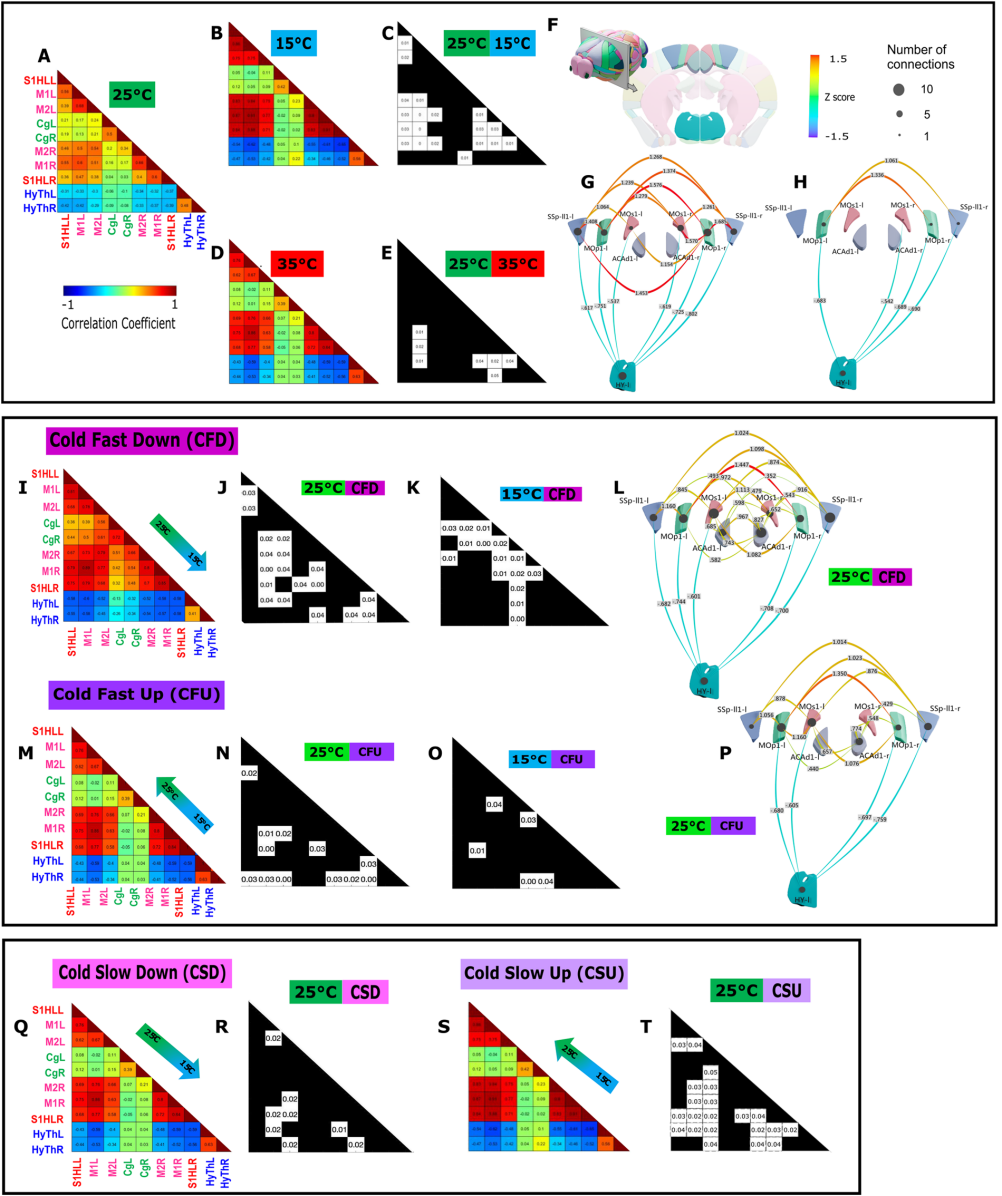
The exposure to a cold floor at constant or varying temperature induces dramatic changes of FC in the somatomotor and hypothalamic networks. ***A–H***, Changes in FC during exposure of the animal to constant floor temperatures (15°C, 25°C, and 35°C); ***I–P***, During exposure to fast cooling temperatures [cool fast down (***I–L***)/cool fast up (***M–P***)] and finally, exposure to cooling at a slower pace of temperature change (***Q–T***). In each experimental condition, the results are presented as an average Pearson correlation matrix of *N* = 6–10 imaging sessions (***A***,***B***,***D***,***I***,***M***,***Q***,***S***), and significance matrices indicating the ROI pairs with significant differences (adjusted *p* < 0.05 indicated in white cells) between two experimental conditions among 15°C, 25°C, 35°C, CFD (cold fast down), CFU (cold fast up), CSD (cold slow down), and CSU (cold slow up). ***F***, Schematic of the imaging plane bregma −0.34 mm in relation to the 3D whole mouse brain and the imaging plane with the studied regions (SSpll1-l/r, primary somatosensory cortex of hindlimb S1HL; MOp1-l/r, primary motor cortex M1; MOs1-l/r, secondary motor cortex M2; ACAd1-l/r, cingulate Cg; and HY l/r, hypothalamus). ***G***, ***H***, ***L***, ***P***, Summary representation of the statistically significant changes of FC within the ROI (shown as *z*-scores) between the conditions: fixed 25°C and 15°C (***G***) and fixed 25°C and 35°C (***H***), cold fast down and 25°C (***L***), and cold fast up and 25°C (***P***). The exact number of sessions and animals per session are as follows: (1) for constant temperatures: *N* = 8 sessions, from *N* = 7 (25°C) or *N* = 5 mice (15°C and 35°C). (2) Ramps: cold fast up and down ramps, *N* = 10 sessions from *N* = 4 mice; cold slow down and warm slow up and down, *N* = 8 sessions from *N* = 3 mice; warm fast down and up, *N* = 9 sessions from *N* = 5 mice; and finally *N* = 6 sessions from *N* = 3 mice for cold slow up. Further details are presented in Extended Data Figure 1-1. Our results show a reinforcement of the somatomotor FC and a dissociation of the SM–hypothalamic FC during exposure to either fixed cold temperature or fast cooling temperature. These changes seem largely unidirectional. Constant temperatures were also compared with 35°C (***D***,***E***,***H***) and also to varying warm temperatures (Extended Data Fig. 3-1), but these elicited very modest changes. Boxplots presenting Pearson’s correlation coefficient for each ROI pair with a significant FC alteration are shown in Extended Data Figures 3-1, 3-2, 3-3, and 3-4.

In the first part, we investigated the potential changes of FC in cortical and hypothalamic areas located in the chosen imaging plane during 20 min of exposure to a constantly neutral (25°C), warm (35°C), or cold (15°C) floor (Fig. 3*A–H*). After removing motion artifacts and concatenating the cleaned signals, the time series were first analyzed in a stationary way, that is, averaged over the recording sessions.

10.1523/JNEUROSCI.0909-23.2023.f3-1 Figure 3-1 **Boxplot representation of ROI pairs with a significant FC alteration between constant 15°C, 25°C and 35°C.** A and C show the significance matrices presented in figure 2. B and D show the coefficient correlation for each pair of significant ROI in the aforementioned pair of ROI. *p < 0.05, **p < 0.01 and ***p < 0.001 of the linear mixed model analysis of the thermal condition effect, followed by Benjamini-Hochberg’s correction for multiple comparisons. Download Figure 3-1, PDF file.

10.1523/JNEUROSCI.0909-23.2023.f3-2 Figure 3-2 **No changes in FC are observed between Warm Fast Up or Warm Down ramps and either 35°C or 25°C conditions using correlation matrices.** (A, B) Averaged Pearson correlation matrices of WFU (N=8) and WFD (N=8) imaging sessions. (C, E). Average Pearson correlation matrix of N=8 imaging sessions at 25°C. No significant FC alteration was observed. Download Figure 3-2, PDF file.

10.1523/JNEUROSCI.0909-23.2023.f3-3 Figure 3-3 **Boxplot representation of ROI pairs with a significant FC alteration between Cool Fast Down (A-D) or Cool Fast up ramps (E-H) and 25°C and 15°C respectively.** A, C, E and F show the significance matrices presented in figure 2. B, D, F and H show the coefficient correlation for each pair of significant ROI in the aforementioned pair of ROI. *p < 0.05, **p < 0.01 and ***p < 0.001 of the linear mixed model analysis of the thermal condition effect, followed by Benjamini-Hochberg’s correction for multiple comparisons. Download Figure 3-3, PDF file.

**Figure 4.**
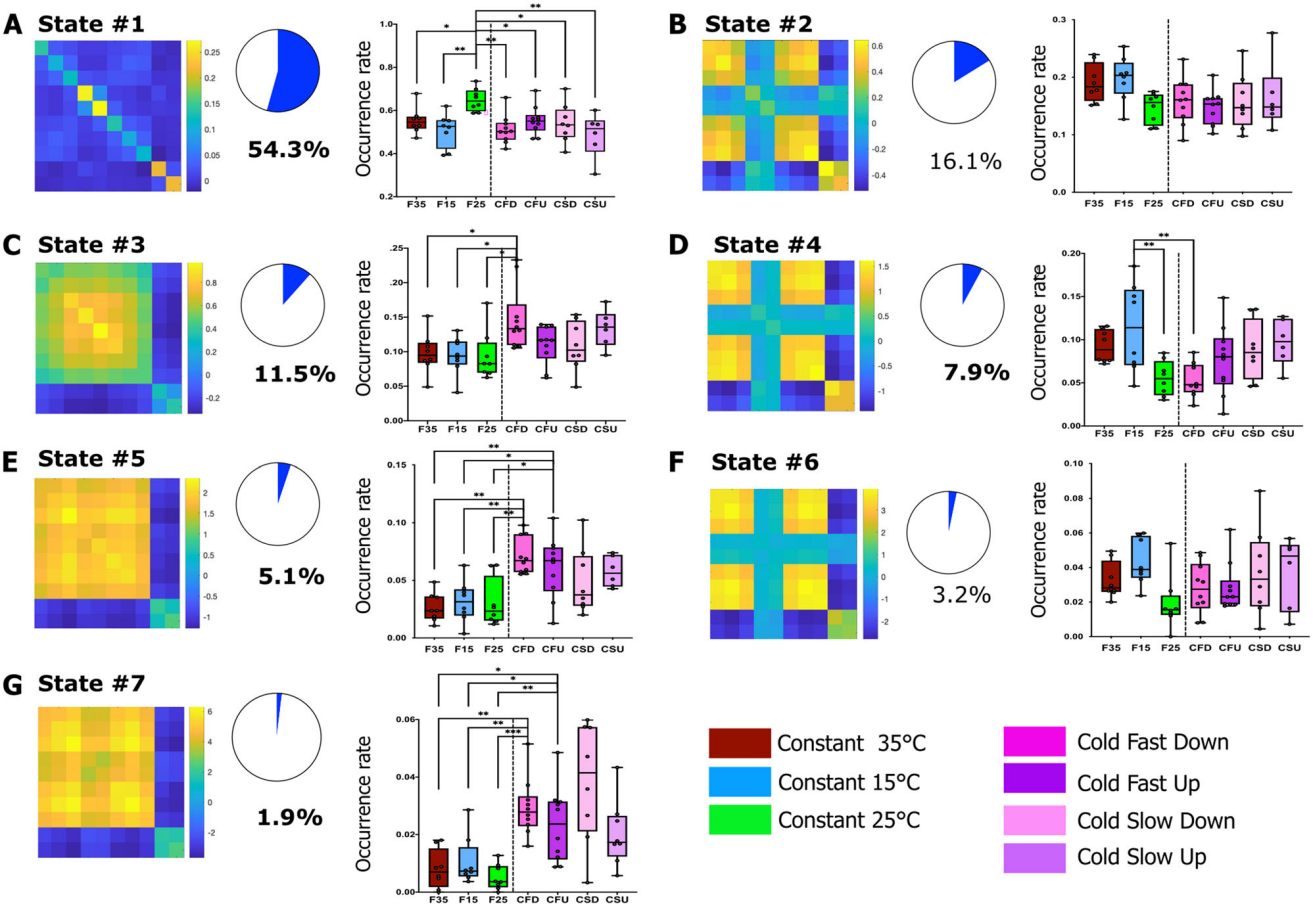
Dynamic FC analysis of thermal coding using *k*-means clustering. Seven brain state matrices (cofluctuation matrices) corresponding to the seven dynamic brain states defined by *k*-means clustering were ordered according to their decreasing occurrence rate (***A–F***). The occurrence rates in all experimental conditions were calculated and compared between experimental conditions using a linear mixed model, the significance being corrected by Benjamini–Hochberg's procedure for multiple comparisons. *p < 0.05, **p < 0.01, and ***p < 0.001. The weakly correlated state #1, which occurs the most in all conditions (54%), is significantly more frequent in the 25°C condition (***A***). Brain states #3, #5, and #7 show a dichotomy between SM/Cg and hypothalamus and occur more often in cold fast down ramps (***C***,***E***,***G***), whereas state #4 predominantly occurs during sustained cold exposure and displays a decreased connectivity between Cg and other regions (***D***). No significant changes were observed between warm ramps and the fixed temperatures (Extended Data Fig. 4-1).

10.1523/JNEUROSCI.0909-23.2023.f3-4 Figure 3-4 **Boxplot representation of ROI pairs with a significant FC alteration between Cool Slow Down (A-D) or Cool Fast up ramps (E-H) and 25°C and 15°C respectively.** A and C show the significance matrices presented in figure 2. B and D show the coefficient correlation for each pair of significant ROI in the aforementioned pair of ROI. *p < 0.05, **p < 0.01 and ***p < 0.001 of the linear mixed model analysis of the thermal condition effect, followed by Benjamini-Hochberg’s correction for multiple comparisons. Download Figure 3-4, PDF file.

Exposure to constant floor temperature, 35°C (warm) and 15°C (cool) temperatures, for 20 min were compared with 25°C (neutral temperature). We observed large and statistically significant differences of FC between these conditions, with the largest number of significant differences for the cold (15°C) floor, for which the FC of 19 couples of ROI were statistically significantly different (Fig. 3*A–C*,*G*). Twelve of these pairs concerned areas of the somatomotor network (SMN), which had a stronger connectivity at 15°C than at 25°C (Extended Data Fig. 3-1B). A single ROI pair, concerning cingulate and hypothalamus, exhibited an increase in connectivity at 15°C. The six other pairs of ROI concerned areas between a ROI of the SMN and the hypothalamus (Extended Data Fig. 3-1B). Interestingly, in these pairs, the FC was altered in the opposite way: the FC was significantly decreased.

Exposure to warm floors (35°C) induced mild changes of the brain FC (Fig. 3*D*,*E*,*H*). Only seven ROI pairs displayed a significant alteration of the FC. Two of these were pairs of the SMN, which displayed an increased FC (Extended Data Fig. 3-1D). The five other ROI pairs concerned areas of the SMN and the hypothalamic nuclei (Extended Data Fig. 3-1D). They all displayed the opposite effect: an increased FC when the mice were submitted to a warmer floor.

To conclude, for both temperatures (15°C and 35°C), when the floor temperature is constant, the somatomotor regions display an increased connectivity, while the hypothalamic–somatomotor network connectivity decreases. Interestingly, the FC is not significantly different between 15 and 35°C. By taking a closer look, it was observed that the connectivity between somatomotor network is higher at 15°C and 35°C than that at 25°C and that the hypothalamic–somatomotor network anticorrelation was also higher at 15°C and 35°C than that at 25°C.

### Alterations of the stationary FC during fast dynamic changes of temperature

In order to decipher some of the central changes that take place during dynamic changes of the temperature, we next compared the stationary FC during fast or slow, warm or cold ramps.

Warm fast ramps did not lead to any significant FC difference compared with 25°C and 35°C conditions (Extended Data Fig. 3-2). However, down and up cold fast ramps led to strong significant FC alterations compared with 25°C and 15°C conditions in numerous ROI pairs (Fig. 3*I–P*). The cold fast ramps down were the conditions that produced the largest number of statistically different ROIs, with 18 pairs of regions modified with respect to the neutral temperature (25°C; Fig. 3*J*; Extended Data Fig. 3-3B), and 15 pairs when compared with the target temperature (15°C; Extended Data Fig. 3-3D).

When compared with 25°C, five pairs of regions involving the cingulate and SMN and seven couples within the SMN displayed an increased FC when the temperature was lowered (Extended Data Fig. 3-3B). The last six ROI pairs concerned the hypothalamus and SMN or the cingulate. They all had an initial anticorrelation at 25°C, which became even stronger when the floor became colder.

The comparison with constant 15°C floor temperature revealed 15 pairs of regions with significantly modified FC (Extended Data Fig. 3-3D). Among them, 13 encompassed the SMN, where the FC increased when the floor temperature decreased. Two (intra-motor network and motor-S1HL) had the opposite behavior: slight but statistically significantly decreased FC. Finally, as shown in panel B of Extended Data Figure 3-3, networks between the hypothalamus and the cingulate showed anticorrelations when the floor was getting colder.

Overall, these results suggest that a fast decrease in temperature is associated with a strengthening of the correlation of the somatomotor network, and a weakening of the link between the hypothalamus and the somatomotor network.

The analysis of the cold fast ramp up (Fig. 3*M–P*) showed a similar effect in a smaller number of regions. Compared with the target temperature (25°C), five pairs of regions within the SMN showed an increased FC during the ramp (Extended Data Fig. 3-3F). Seven couples involving the hypothalamus and areas of the SMN displayed an increased anticorrelation of their FC. The comparison of this fast-cold up ramp to the initial temperature (15°C) revealed that only six pairs of regions (Extended Data Fig. 3-3H) differed significantly (four within the SMN and two between the hypothalamus and the cingulate and motor cortex).

The striking difference between the two cold fast ramps (down and up) was the large number of subnetworks within the SM–cingulate networks in which the FC was reinforced. In both cases (up and down), the hypothalamus–SM–cingulate networks which are slightly anticorrelated in neutral conditions display a stronger anticorrelation during these ramps (Fig. 3*L*,*P*). These results highlight the complex inter-regional FC between the cingulate, somatosensory and motor cortices, and hypothalamus during exposure to cold.

### Effect of the rate of temperature change

In order to further understand how changes of floor temperature are processed centrally, we also investigated the effect of the temperature change at a smaller rate of 0.5°C/min (20 min duration), called cold slow/warm up/down, that we compared with the fast ramp initially used (1°C/min, 10 min duration). We observe that, as hypothesized, these contrasting conditions produce strikingly different effects. As a matter of fact, the warm slow ramps only lead to slight FC alterations (Extended Data Fig. 3-4).

Only eight pairs of ROI were significantly different between the cold slow down ramp and constant 25°C (Extended Data Fig. 3-4B), which is roughly half of what we observed with the fast equivalent of this ramp. As observed with the fast-cold ramp, the modified ROI pairs concerned the SMN (in which the FC was reinforced), and the SMN and hypothalamus, where the anticorrelation was also reinforced.

As for the cold up ramp, in contrast with the aforementioned cold slow down ramp and very similarly to the fast ramps, it displayed a large number of pairs of ROI modified. Most of them were within the SMN (12 out of 21; Extended Data Fig. 3-4D) with a strengthening of the FC between them. The anticorrelation between the hypothalamus and the SMN was significantly reinforced here.

### Dynamic FC

The dynamic nature of the stimuli applied in this study and the rapid changes of brain states occurring in awake and conscious animals naturally led to the analysis of the dynamic connectivity. We predicted that the FC changes during thermal variations might be even stronger than the differences between warm and cold conditions.

In a previous study, we established that fUS imaging can robustly identify brain states extracted from *k*-means clustering of steady-state fUS data in anesthetized rats (Rahal et al., 2020). In the current study, using a similar approach, but adapted to data discontinuity generated by artifact removal, we identified seven brain states using *k*-means clustering (Fig. 4). After ordering them by their occurrence rate across all animals and experimental sessions, the occurrence rate of the brain states was compared between experimental conditions. This analysis showed that among the seven states, only five had different occurrence rates between some conditions. The states #2 and #6 were homogeneously observed in all conditions. The remaining five states can be grouped in three groups, due to their similarity of behavior.

10.1523/JNEUROSCI.0909-23.2023.f4-1 Figure 4-1 **Lack of statistical difference in the occurrence rate of the various dynamic FC states in warm experiments (constant and ramps).**  As presented in figure 3, dynamic FC was analyzed using k-means clustering with k= 7, which are ordered from 1 to 7 in the order of decreasing overall occurrence rate, i.e. for all animals in all thermal conditions. No significant difference could be established using a linear mixed model analysis of the effect of the thermal condition. Download Figure 4-1, PDF file.

The first group comprises state #1 only, which is by far the most frequent state (54–60% of the time; Fig. 4*A*), has weak connections, and is consistently more present at neutral temperature (constant 25°C) compared with all other experimental conditions (Fig. 4*A*).

The second group is made up of states #3, #5, and #7 (which are present 10–17% of the time) are characterized by a common pattern of connectivity in the SM–cingulate (SM–Cg) cortex and an anticorrelation between the SM and the hypothalamic regions. Interestingly, these three states were significantly more frequent during the CFD ramp, compared with all constant temperatures.

The third group consisted of state #4 only. In addition to the dichotomy between the SM and hypothalamic networks, these states (present in 10–13% of the time) are characterized by a low correlation between the cingulate and all other areas. Very interestingly, it is consistently more frequent in only one condition, the low constant temperature (15°C), suggesting a specific involvement of the cingulate cortex during this prolonged (20 min) exposure to cold.

Finally, the occurrence of these brain states was never significantly modified during exposure to warm ramps (Extended Data Fig. 4-1).

## Discussion

The study presented here delves into the intricate web of functional connectivity in mice, with a particular focus on how these networks respond to thermal stimuli.

### FC: a strong marker of strength of interaction between brain regions

The analysis of intrinsic brain activity has led to the definition of resting brain state networks. Since then, the field of neuroscience has been widely studying these infraslow oscillations, called FC, that characterize the spatiotemporal organization and function of large-scale brain networks (Preti et al., 2017). Interestingly, FC can be studied using very different approaches, such as fMRI, electrophysiology, MEG, fiber photometry, optical imaging, and fUS (Pais-Roldán et al., 2021). Although the classical studies focused on resting-state FC, task- or behavior-related FC studies using fMRI have also been increasing in numbers in the recent years (Barch et al., 2013; Cole et al., 2014; Di and Biswal, 2019). Although the mice are not subjected to a task in our study, they are physiologically and behaviorally responding to the thermal stimulation which is either cold, warm, or neutral.

### Warm conditions do not elicit FC alterations

The temperature conditions in the present study were carefully designed as not to induce any discomfort in the mice during awake imaging. Although 25°C falls in a slightly low temperature range, adaptation takes place before the beginning of the experiment and can be considered neutral. Thermal psychophysics studies have shown that, when the skin is adapted to temperature values ranging from ∼30 to ∼34°C, neither warm nor cool sensations are experienced (Filingeri, 2016). Furthermore, the transition from ambient to high temperature between 32–39°C and 26–34°C activates the TRPV3 and TRPV4 channels, respectively. Therefore, it is likely that a temperature of 35°C would not either have evoked a significantly different response from that observed at neutral temperature.

### Dichotomy between somatomotor–cingulate and hypothalamic networks during exposure to cold

In neutral conditions, the stationary FC indicated that, as previously demonstrated in fMRI in human (Zeng et al., 2012) and rodents (see for review Pais-Roldán et al., 2021), the SMN has a strong positive interhemispheric connectivity that was shown previously to be due to a strong concomitant interhemispheric neuronal activity. Our study demonstrates a reinforcement in the somatomotor FC during cold sensing, mostly in the cold fast ramps. We hypothesized that such an FC increase was an indirect readout of the sensory discriminative aspect of cold sensing, a feature recently described at the cellular level using calcium imaging (Vestergaard et al., 2023).

In contrast, the SM–hypothalamic network shows a decreased FC during cold sensing, suggesting a dichotomy between these two networks. A similar dramatic opposing effect was also observed when analyzing the dynamic FC. Indeed, the second type of dynamic modes reproducibly obtained in our analysis was the states 3, 5, and 7, which are characterized by a dichotomy between a strongly correlated signal within the SM–Cg network and an anticorrelation in the SM–hypothalamic network. These three modes, which account for ∼10–17% of the time, have a higher occurrence during the cold fast down ramp. The significant differences with other conditions, including the cold slow down ramps and the constant cold condition (15°C), suggest that this dichotomy is a specific feature of cold sensing.

### Weakly connected dynamic mode in thermal sensing

By measuring the temporal fluctuations in FC, using *k*-means clustering, we identified seven dynamic patterns of fluctuations. These seven states encompass different neurobiological meaning and role.

The first group of states consists of state #1 alone. As previously described in monkeys (Demertzi et al., 2019) and human subjects (Demertzi et al., 2019) using the same type of analysis but in fMRI, the most frequent dynamic state (50–60% of the recording time) is a mode characterized by weak strengths of connection. Our current understanding of this state is that it is associated with low-level cognitive functions (Demertzi et al., 2019). In our study, while present in the majority of all experimental groups, it is statistically more frequent when the animals were exposed to the neutral temperature of 25°C. This result reinforces previous suggestions of low FC, apart from the default mode network during resting periods.

### A secondary pathway involving hypothalamus

The hypothalamus was anticorrelated to the somatomotor network in all conditions, and the strength of this anticorrelation was the highest during cold sensing. This dramatic effect was revealed in both stationary and dynamic FC analysis. Although the preoptic anterior hypothalamus (POAH) has been linked to thermoregulatory behavior (Ishiwata et al., 2002; Wang et al., 2019), the secondary thermosensory pathway from the dorsal horn to the lateral parabrachial nucleus (LPB) and then to the preoptic area (POA) of the hypothalamus has been put forward by Yahiro et al. (2017). Using selective lesions of either thalamic nuclei or the POA and the LPB, they unraveled an important role of these nuclei in thermoregulatory behaviors (Yahiro et al., 2017). Our hypothesis is that the changes observed in the hypothalamus in our study are linked to this role in thermoregulation.

Only a small number of FC studies in rodents are documenting networks involving hypothalamic nuclei. In the rare cases doing so, they define them as the “ventral midbrain” (Liska et al., 2015) or the “basal ganglia-hypothalamus” (Becerra et al., 2011). In agreement with our observations, these networks are different from the sensory network (Hutchison et al., 2010; Zerbi et al., 2015). If we postulate that this anticorrelation is due to a decrease in neuronal activity, this second structure would have an inhibitory action on hypothalamic neurons. However, this anticorrelation can also be due to a time lag between signals of the SMN and the hypothalamic. In this last case, the hypothesized structure(s) would only change the temporal shift, without affecting the intensity of neuronal activity.

### Sustained cold and cold ramps are encoded differentially in the cingulate cortex

During fast cooling, interhemispheric connectivity within the cingulate cortices increased significantly, as well as the connectivity between the SM and the cingulate cortices. When the cold ramp was slow, however, the FC between these regions was not affected. The thermal psychophysics associated with rapid temperature changes and the already established role of the cingulate cortices in affective responses to unpleasant or nociceptive thermal sensations (Craig et al., 1996; Becerra et al., 1999; Brooks et al., 2002; Derbyshire et al., 2002) has led us to consider that the concomitant FC increase between SM and cingulate cortex suggests a common hub between the two.

In the dynamic brain states #4 and #6, on the other hand, the cingulate cortex is differently connected to the other networks during static exposure to a lower temperature (15°C). The decrease in connectivity with the somatomotor network in this state seems to be a characteristic of sustained exposure to cold, and this suggests the differential role of cingulate in sensing persistent cold sensations and cold ramps.

### Limitations of this study and future work

The field of thermal sensing has benefited from the advent of various approaches, such as electrophysiological recordings of dissociated neurons (Caterina et al., 1999) and behavioral tests assessing warm/cold sensitivity, (Bautista et al., 2007; Touska et al., 2016; Masuda et al., 2022) or operant thermal perception task (Milenkovic et al., 2014). Used alone in genetically modified mice (Kasuga et al., 2022) or in combination with a direct measure of neuronal activity (Milenkovic et al., 2014), these studies have offered invaluable information about the role of certain ion channels in thermal sensation.

In vivo calcium imaging, thanks to its ability to measure neuronal activity, has led light on the differential coding properties for warming/cooling temperatures of sensory neurons (Wang et al., 2018), a phenomenon not observed in the spinal cord (Ran, 2016). At the cortical level, large-scale imaging revealed clear differences in the respective involvement of the primary sensory cortex and the insular cortex in encoding cool/warm sensations (Vestergaard et al., 2023).

fUS imaging is a relatively new technique, still under development, especially for whole-brain imaging (Rabut et al., 2020; Demeulenaere et al., 2022). Due to this technical limitation, our study could not discover new brain areas involved in thermal sensing. Instead, we took full advantage of its high temporal resolution and wide field of view to study the concomitant dynamics of signals in regions not previously studied simultaneously. With the introduction of new probes for three-dimensional ultrasound imaging, it will be possible in the near future to use fUS imaging to functionally define new brain regions involved in thermal sensing. Additionally, the use of genetically modified mice will be particularly interesting for deciphering the specific roles of particular channels in various physiological parameters modified during temperature changes.
