# Supplementary material for: Specific and Nonuniform Brain States during Cold Perception in Mice
Source: J Neurosci. 2024 Jan 5;44(12):e0909232023. doi: 10.1523/JNEUROSCI.0909-23.2023 (PMC10957214; doi:10.1523/JNEUROSCI.0909-23.2023)
Supplement: Figure 1-1 — Tables reporting the identity of the mice included in each set of experiments, as well as the number of imaging sessions kept per animal in our analysis. Download Figure 1-1, PDF file. [file jneuro-44-e0909232023-s001.pdf]

I. Number of imaging sessions kept in constant temperature experiments

Nb Mouse	Constant 25°C	Constant 15°C	Constant 35°C
M401	1	1	1
M402	1	2	1
M403	2	2	2
M404	1	2	2
M405			1
M406	1	1	1
M302	1		
M304	1		
Total sessions	8	8	8

II. Number of imaging sessions kept in Ramp experiments

	Cold Fast Down	Cold Fast Up	Cold Slow Down	Cold Slow Up
M501	3	3	3	3
M502	3	3		
M503	2	2	2	1
M504				
M505	2	2	3	2
M506				
Total sessions	10	10	8	6

	Warm Fast Down	Warm Fast Up	Warm Slow Down	Warm Slow Up
M501	3	3	3	3
M502	2	2		
M503	2	2	2	2
M504				
M505	2	2	3	3
M506				
Total sessions	9	9	8	8

Extended figure 1-1: Summary presenting the mice (M) included in each part of the study and the number of sessions kept for each one of them. The numbers indicate how many sessions of each mouse was kept.The number following ‘M’ is the mouse number. Ex/ M401: Mouse #401.Grey boxes: due to artifacts, the signals obtained for this recording was too noisy and had therefore to be discarded (see materials and methods).
