# Supplementary material for: Specific and Nonuniform Brain States during Cold Perception in Mice
Source: J Neurosci. 2024 Jan 5;44(12):e0909232023. doi: 10.1523/JNEUROSCI.0909-23.2023 (PMC10957214; doi:10.1523/JNEUROSCI.0909-23.2023)
Supplement: Figure 3-2 — No changes in FC are observed between Warm Fast Up or Warm Down ramps and either 35°C or 25°C conditions using correlation matrices. (A, B) Averaged Pearson correlation matrices of WFU (N=8) and WFD (N=8) imaging sessions. (C, E). Average Pearson correlation matrix of N=8 imaging sessions at 25°C. No significant FC alteration was observed. Download Figure 3-2, PDF file. [file jneuro-44-e0909232023-s003.pdf]

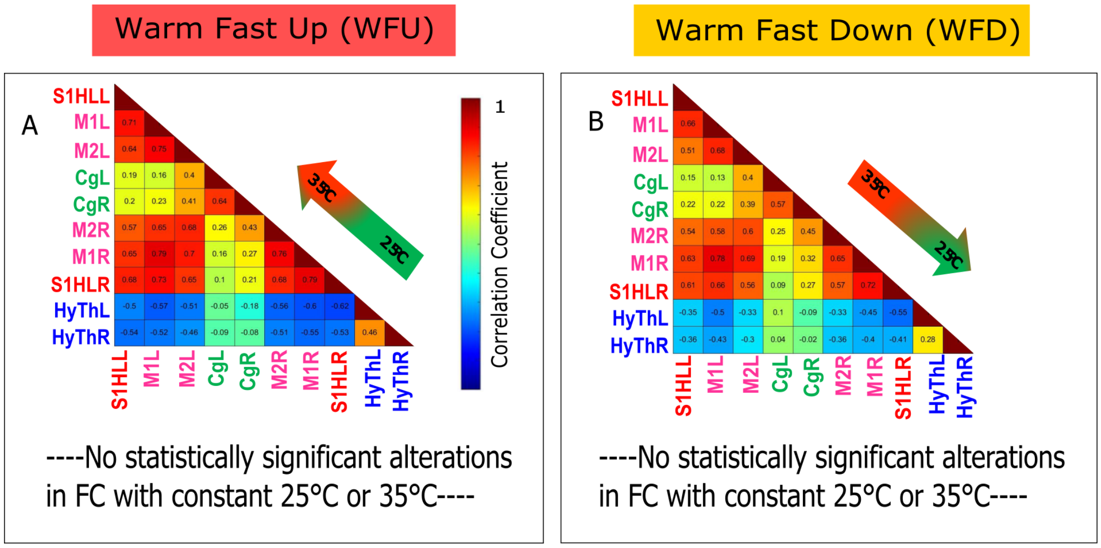


Extended figure 3-2: No changes in functional connectivity are observed between Warm Fast Up, Down ramps and either 35°C or 25°C conditions using correlation matrices. (A, B) Averaged Pearson correlation matrices of WFU (N=8) and WFD (N=8) imaging sessions. No significant FC alteration was observed.
