# Supplementary material for: Specific and Nonuniform Brain States during Cold Perception in Mice
Source: J Neurosci. 2024 Jan 5;44(12):e0909232023. doi: 10.1523/JNEUROSCI.0909-23.2023 (PMC10957214; doi:10.1523/JNEUROSCI.0909-23.2023)
Supplement: Figure 3-3 — Boxplot representation of ROI pairs with a significant FC alteration between Cool Fast Down (A-D) or Cool Fast up ramps (E-H) and 25°C and 15°C respectively. A, C, E and F show the significance matrices presented in figure 2. B, D, F and H show the coefficient correlation for each pair of significant ROI in the aforementioned pair of ROI. *p < 0.05, **p < 0.01 and ***p < 0.001 of the linear mixed model analysis of the thermal condition effect, followed by Benjamini-Hochberg’s correction for multiple comparisons. Download Figure 3-3, PDF file. [file jneuro-44-e0909232023-s004.pdf]

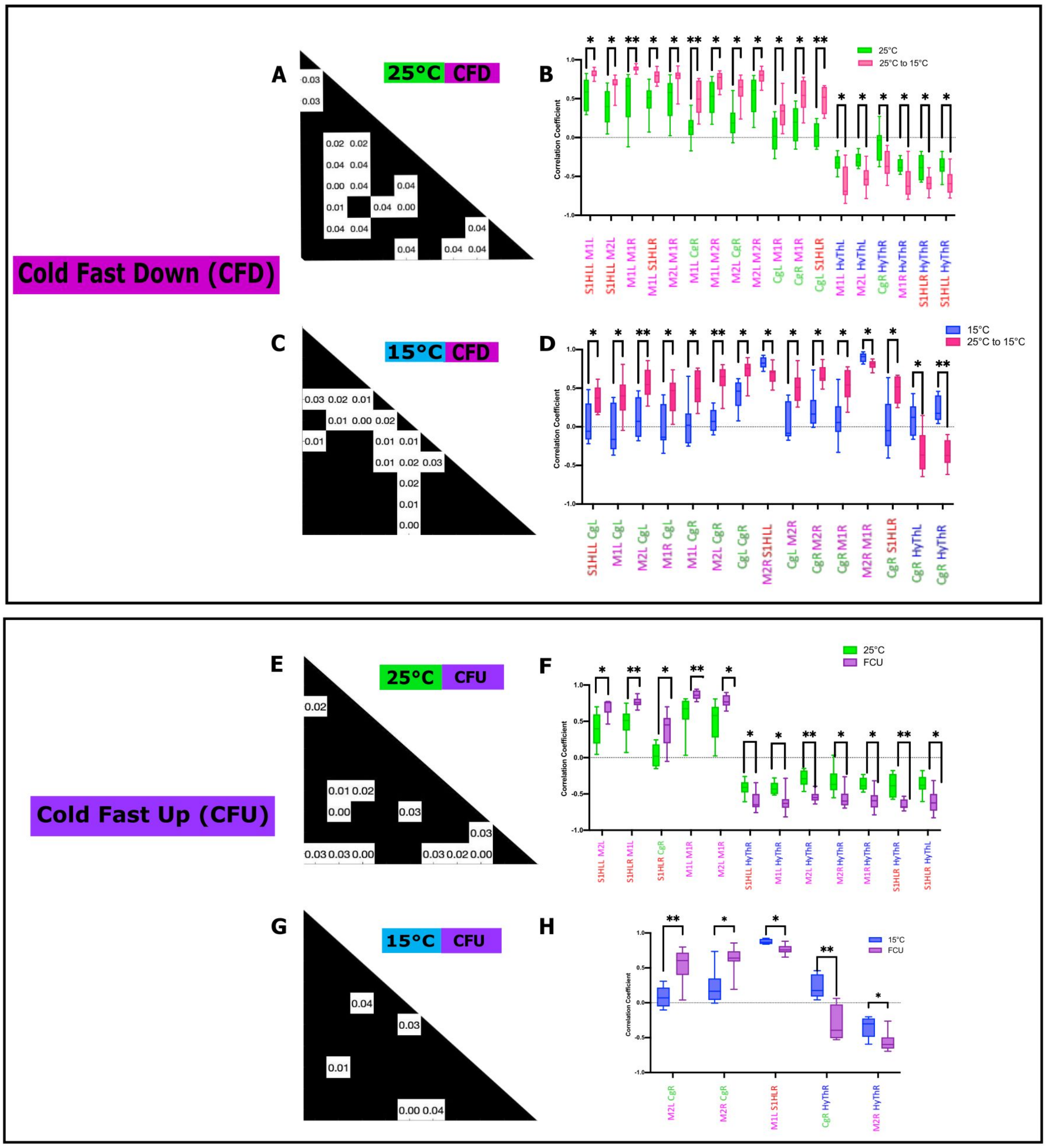

Extended figure 3-3: Boxplot representation of ROI pairs with a significant FC alteration between Cool Fast Down (A-D) or Cool Fast up ramps (E-H) and 25°C and 15°C respectively.

A, C, E and F show the significativity matrices presented in figure 2. B, D, F and H show the coefficient correlation for each pair of significant ROI in the aforementioned pair of ROI. *p < 0.05, **p < 0.01 and ***p < 0.001 of linear mixed model analysis of the thermal condition effect, followed by Benjamini-Hochberg’s correction for multiple comparisons.
