# Supplementary material for: Specific and Nonuniform Brain States during Cold Perception in Mice
Source: J Neurosci. 2024 Jan 5;44(12):e0909232023. doi: 10.1523/JNEUROSCI.0909-23.2023 (PMC10957214; doi:10.1523/JNEUROSCI.0909-23.2023)
Supplement: Figure 4-1 — Lack of statistical difference in the occurrence rate of the various dynamic FC states in warm experiments (constant and ramps). As presented in figure 3, dynamic FC was analyzed using k-means clustering with k= 7, which are ordered from 1 to 7 in the order of decreasing overall occurrence rate, i.e. for all animals in all thermal conditions. No significant difference could be established using a linear mixed model analysis of the effect of the thermal condition. Download Figure 4-1, PDF file. [file jneuro-44-e0909232023-s006.pdf]

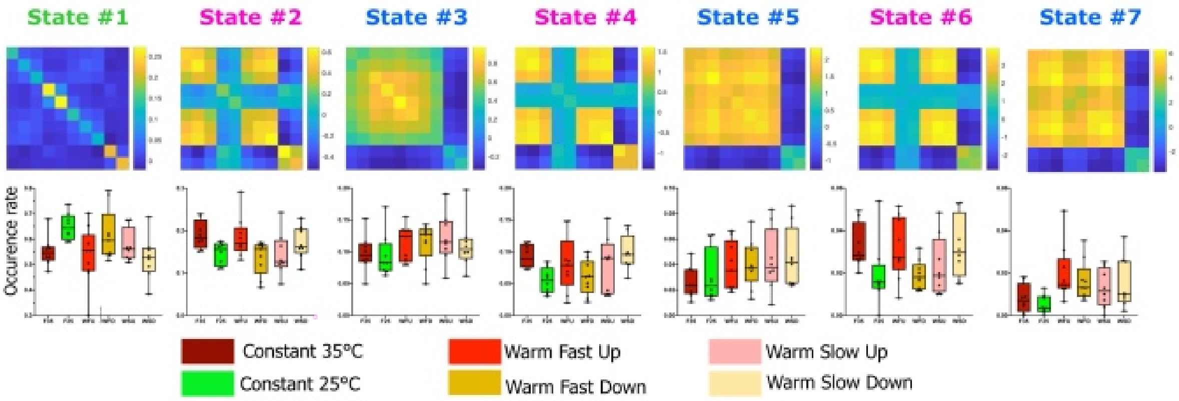

Extended figure 4-1: Lack of statistical difference in the occurrence rate of the various dynamic functional connectivity states in warm experiments (constant and ramps).
As presented in figure 4, dynamic functional connectivity was analyzed using k-means clustering with k= 7, which are ordered from 1 to 7 in the order of decreasing occurrence rate. The frequency of occurrence was calculated for each brain state for all thermal conditions. No significant difference could be established using a linear mixed model analysis of the thermal condition.
