# Supplementary material for: Specific and Nonuniform Brain States during Cold Perception in Mice
Source: J Neurosci. 2024 Jan 5;44(12):e0909232023. doi: 10.1523/JNEUROSCI.0909-23.2023 (PMC10957214; doi:10.1523/JNEUROSCI.0909-23.2023)
Supplement: Figure 3-1 — Boxplot representation of ROI pairs with a significant FC alteration between constant 15°C, 25°C and 35°C. A and C show the significance matrices presented in figure 2. B and D show the coefficient correlation for each pair of significant ROI in the aforementioned pair of ROI. *p < 0.05, **p < 0.01 and ***p < 0.001 of the linear mixed model analysis of the thermal condition effect, followed by Benjamini-Hochberg’s correction for multiple comparisons. Download Figure 3-1, PDF file. [file jneuro-44-e0909232023-s002.pdf]

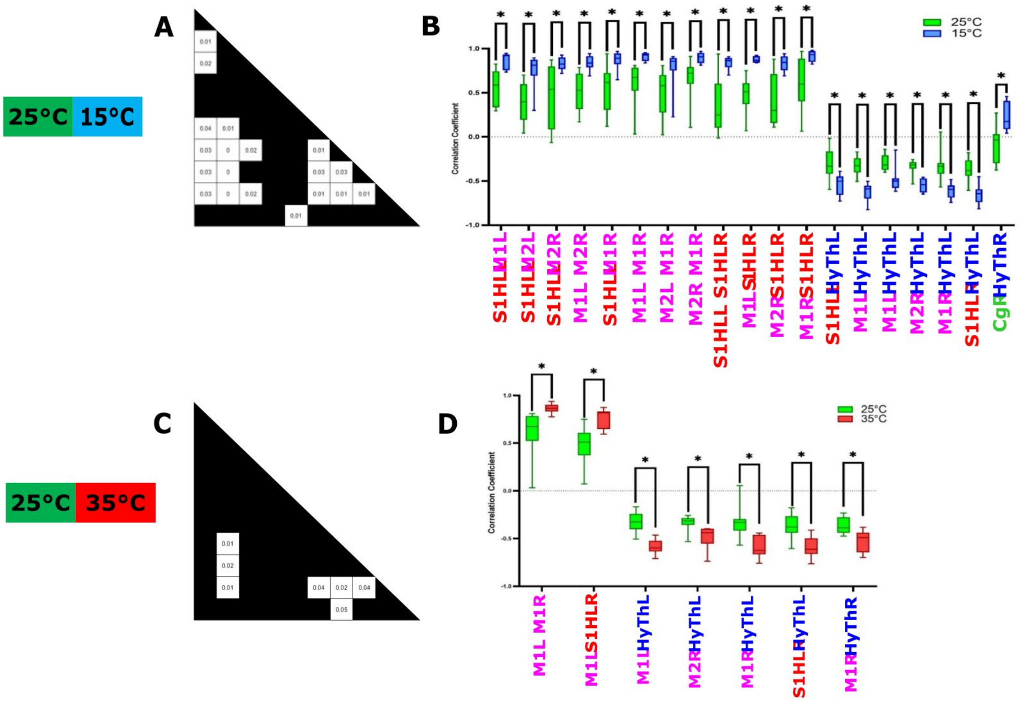

Extended figure 3-1: Boxplot representation of ROI pairs with a significant FC alteration between constant 15°C, 25°C and 35°C. A and C show the significativity matrices presented in figure 2. B and D show the coefficient correlation for each pair of significant ROI in the aforementioned pair of ROI. *p < 0.05, **p < 0.01 and ***p < 0.001 of linear mixed model analysis of the thermal condition effect, followed by Benjamini-Hochberg’s correction for multiple comparisons.
